# Low salinity water flooding: estimating relative permeability and capillary pressure using coupling of particle swarm optimization and machine learning technique

**DOI:** 10.1038/s41598-024-61168-2

**Published:** 2024-06-08

**Authors:** Razieh Khosravi, Mohammad Simjoo, Mohammad Chahardowli

**Affiliations:** Faculty of Petroleum and Natural Gas Engineering, Sahand University of Technology, Tabriz, Iran

**Keywords:** Chemical engineering, Mechanical engineering

## Abstract

The reservoir’s properties are required for proper reservoir simulation, which also involves uncertainties. Experimental methods to estimate the relative permeability and capillary pressure data are expensive and time-consuming. This study aims to determine the relative permeability and capillary pressure functions of a sandstone core in the presence and absence of clay during low-salinity water floods. The data were provided by automatic history matching the results from previously lab-reported studies through coupling a simulator with the particle swarm optimization algorithm. Correlations were proposed using multiple-linear regression for relative permeability and capillary pressure parameters at low-salinity conditions. They were validated against experimental results of no clay and clayey formation with regression of 95% and 97%. To assign one curve of relative permeability and capillary pressure to the grid cells of the simulator, averaging techniques were implemented. The effect of salinity and clay content on the obtained curves was investigated. Changing salinity from 42000 to 4000 ppm, the reduction in water relative permeability appeared to be higher than the oil relative permeability increment. Moreover, a noticeable shift in the relative permeability curves toward the highest saturations related to the clay content was observed. The proposed hybrid method could be a suitable tool to estimate the relative permeability and capillary pressure functions of the water-based EOR methods.

## Introduction

A large amount of the oil reserves remains as residual oil, even using secondary recovery methods like water flooding. Thus, investigating enhanced oil recovery (EOR) techniques to improve oil recovery is very important. Experimental and field evidence demonstrates that low-salinity flood in sandstones improves oil recovery by altering the wettability of the reservoir rock towards a more water-wet state^1^. Many studies in recent years have focused on low-salinity water (LSW) flood as an EOR method for increasing oil recovery in sandstone oil reservoirs^2,3^. A better understanding of the reservoir through developed models leads to more accurate decisions to optimize field applications and increase reservoir productivity^4,5^. Reservoir simulation is the most used tool to realize the prediction of the field^6,7^. The reservoir’s static and dynamic properties throughout the reservoir are required for proper reservoir simulation, which also has uncertainties. As a result, a discrepancy occurs between the simulated and observed values^7^.

History Matching (HM) is a technique where reservoir data matches uncertain parameters of the reservoir model for further reservoir applications^4^. Computer-aided optimization techniques for HM purposes, known as Automatic History Matching (AHM), have been widely applied^5^. It is more efficient than manual methods because it uses an optimization algorithm to find the best values such that the match to dynamic data is acceptable^4,8^. Various optimization methods have been adopted in the AHM process, including Gradient-Based, Hybrid, Stochastic (Genetic Algorithm (GA), Particle Swarm Optimization (PSO)), and Probabilistic methods. Each method has positives and negatives^4,5,8–13^. Many studies aim to find the best HM algorithm among several available methods. Ensemble Kalman Filter (ENKF) and stochastic methods are the most popular algorithms for reservoir HM^4,11,12,14^. ENKF has many advantages, such as the ease of implementation, ease of formulation, and ability to handle non-linear systems. However, it has a high computational cost, and it is suggested for large systems and field optimizations rather than core scale simulations^12^. Both PSO and GA algorithms are suitable for solving complex problems and have easy implementation in every simulator. However, they may not be suitable for large systems, and the problem of convergence speed exists. Based on the purpose of our study, in which HM is conducted at a core scale (ENKF is not necessary) and running time is important, PSO is preferred. The reason is that the optimized parameters will be determined faster and less scattered than GA^10,12^.

Relative permeability curves describe oil and water mobility in the simulation model and are important for successful reservoir simulation^15,16^. Capillary pressure is also significant for LSW flooding because part of the benefit of LSW flooding is to make capillary pressure less negative, and thereby improving water flood efficiency. Therefore, a low-salinity capillary pressure is needed to estimate low-salinity benefits at high resolution properly^17^. Relative permeability and capillary pressure curves are widely applied in reservoir engineering applications^12^, so accurate estimations of them are necessary^12,18^. Since experimental methods to estimate the relative permeability are expensive and time-consuming^12,19,20^, optimization algorithms to estimate these parameters are suggested. In this case, evolutionary and stochastic algorithms could be a promising solution for different applications. For the application of LSW flood in sandstone reservoirs, many studies have been conducted to determine optimization parameters^12,21–23^. Among them, optimization studies to determine relative permeability and capillary pressure functions using the combination of evolutionary and machine learning algorithms are rare.

Flow properties are a function of pore geometry, and each lithology usually has special relative permeability and capillary pressure curves, which are not always available for each rock type^24^. Capillary pressure and relative permeability curves measured in the laboratory may be only available for a subset of samples^25–27^. Although these curves may be available on core scale models, it is required to upscale these core-size curves measured by laboratory experiments for different types of simulation. In other words, several pairs of relative permeability curves should be available because of the uncertainty associated with relative permeability data. The relative permeability and capillary pressure parameters (estimated by laboratory tests or AHM) must be averaged at the core scale to obtain a single pair of curves for each rock type. Coats was the first to introduce pseudo-relative permeability curves based on the assumption of vertical equilibrium in each grid cell of the reservoir model^28^. After that, different methods have been proposed to estimate the average relative permeability (Kyte & Berry, Pore Volume Weighted, Stone, Total Mobility, Quasi-Steady State, and Weighted relative permeability)^29–36^. J-function is the most common method for averaging capillary pressure curves^29,37–40^.

In this study, hybrid particle swarm optimization coupled with a machine learning technique is proposed to determine the relative permeability and capillary pressure functions during the low-salinity water injection for different rock types (with/without clay content) of a heterogeneous sandstone core. To this end, the particle swarm optimization algorithm is coupled with a reservoir simulator to provide the required data by automatic history matching recovery factor and pressure drop results. Correlations are proposed to estimate core scale relative permeability and capillary pressure functions at low-salinity conditions based on multiple linear regression. Different relative permeability and capillary pressure curves were determined using experimental data under different rock and fluid conditions. Then, one single curve was assigned to the grid cells through averaging techniques for core scale low-salinity flood simulation. The effect of salinity and clay content on the obtained relative permeability and capillary pressure curves was investigated. The proposed hybrid method could be a suitable tool for low-salinity flooding applications at the core and field level.

## Method

In this section, the properties of a sandstone core, hybrid PSO coupled with multiple linear regression to provide the required data for proposing correlations, and a discussion on averaging techniques to assign a single capillary pressure and relative permeability curve to grid blocks in the simulator is elaborated.

### Sandstone core properties

**Figure 1:**
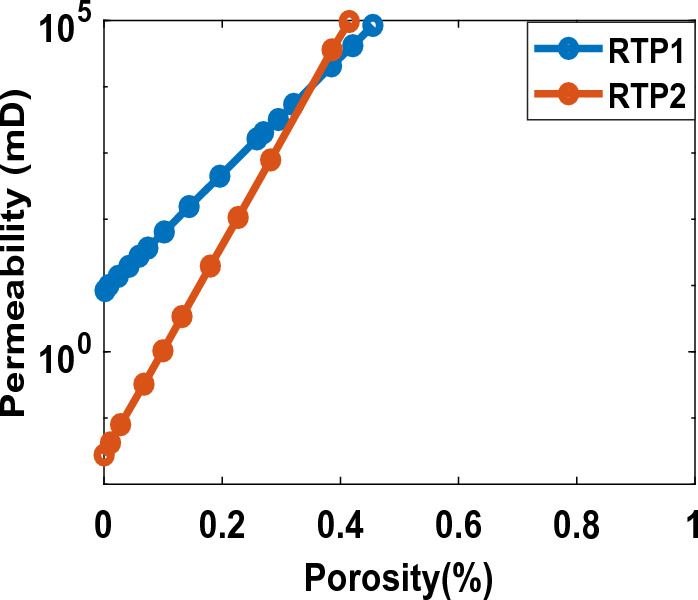
The permeability and porosity correlation for RTP1 and RTP2.

The sandstone core properties are investigated regarding porosity, permeability, clay content, and wettability. Two rock types are selected, and their properties are reported in Table 1:

For RTP1, the average core porosity and permeability are reported as 0.3 and 4.5 (Darcy), respectively. For RTP2, the reported values are 0.20 and 1.50 (Darcy), respectively. RTP1 is considered a no clay formation, and RTP2 is a clayey formation. The permeability and porosity correlation for RTP1 and RTP2 is as shown in Fig. 1:

### Hybrid particle swarm optimization coupled with machine learning technique for estimating core scale relative permeability and capillary pressure curves

To determine the relative permeability and capillary pressure functions for the LSW process at the core scale, the studies of similar sandstone oil reservoirs are used and categorized into different rock types. These parameters are reported in some studies, but most are not. So, there is a need to determine the matching parameters by coupling the reservoir simulator and PSO algorithm.

Studies similar to our lithology (with and without clay content) are gathered thoroughly. Their properties including formation type, injected brine salinity, wettability, and clay content are investigated and the objective of each study is elaborated. The results are summarized in Tables 2 and 3:

**Table 1:** Properties of Rock Type1 and Rock Type2.

Rock type	k-φ relation	Wettability	Clay content
Rock Type1 (RTP1)	Figure 1	Mixed Wet	< 1 wt% (No Clay)
Rock Type2 (RTP2)	Figure 1	Weakley Water Wet	6 wt% (Clayey Formation)

**Table 2:** Studies similar to RTP1 and their properties.

Refs.	Formation	Injected Brine Salinity (ppm)	Wettability	Clay content (%)	Objective
Zhang^41^	Berea Sandstone	387	Mixed to Weakly Water Wet	Kaolinite > Ilite > Chlorite	Mixed wet cores were used to investigate the effect of initial water saturation on secondary and tertiary oil recovery factors
Rivett^42^	Berea Sandstone	1140	Mixed Wet	Less than 5 wt% clay	Mixed wet cores were used to study the effect of LSWF on oil recovery, residual oil saturation, and relative permeability
Winoto^43^	Brial Hill (Sandstone)	17,000	Mixed to Weakly Water Wet	Kaolinite and Ilite	17 mixed wet sandstone outcrops were tested for water flood response to on- twentieth dilution of se water and the oil recovery factor for each case was monitored
Wang^44^	Minnelusa Formation Rock Samples	2800	Mixed Wet	–	Measurements of drainage, spontaneous imbibition, and forced imbibition capillary pressure was analyzed under LSWF conditions
Tahir^3^	Bentheimer Sandstone Core	4000	Mixed wet	Negligible amount of clay (< 1 wt%),	The study focuses on the influence of the sulfate ion on LSWF for applications in sandstone reservoirs
Hernández^45^	Sandstone Core	1000	Mixed wet	–	The impact of salinity on oil recovery and polymer rheology was studied

**Table 3:** Studies similar to RTP2 and their properties.

Refs.	Formation	Injected Salinity (ppm)	Wettability	Clay content (%)	Objective
Kozaki^46^	Berea Sandstone	1000	Mixed Wet	kaolinite, illites, and mixed-layer montmorillonite-illiltes (5 wt%)	Several core flood experiments were conducted to study the efficiency of LSWF in mixed-wet Berea sandstone cores
Aladasani^47^	Berea and Wyoming Sandstone	2500	Mixed wet	–	The sensitivities of LSWF recovery mechanisms in sandstone reservoirs have been measured
Fjelde^48^	Sandstone Reservoir in the North Sea	105	Mixed wet	13 wt%	The brine-rock interactions at high and low salinity conditions using reservoir core plugs were described
Mjøs^49^	Berea Sandstone	3000	Mixed wet	6 wt% kaolinite and 1wt% Ilite	The effect of LSW in secondary and tertiary modes in six Berea cores was compared
Sorop^1^	High Permeability Sandstone Reservoir	1500	Mixed to Weakly Water Wet	–	A steady-state experimental procedure was implemented to measure relative permeability data during LSWF in sandstone core floods
Shojaei^50^	Asmari Formation, from Zagros Fold and Thrust belt	3500	Mixed wet	Ilite > Montmorillonite > Chlorite > Kaolinite	The capillary pressure and relative permeability curves are evaluated from inverse modeling of the obtained pressure drop and oil production data. Then, capillary pressure and relative permeability models as a function of water saturation and salinity are determined
Kadeethum^51^	Sandstone Core	700	Mixed Wet	–	A comparison of LSWF and conventional water flood regarding oil recovery using mixed wet sandstone core plugs was conducted
					A numerical simulation was used to investigate the impact of physical diffusion/dispersion on oil
Attar^52^	Sandstone Core	1000	Weakly Water Wet	–	recovery from LSWF

**Figure 2:**
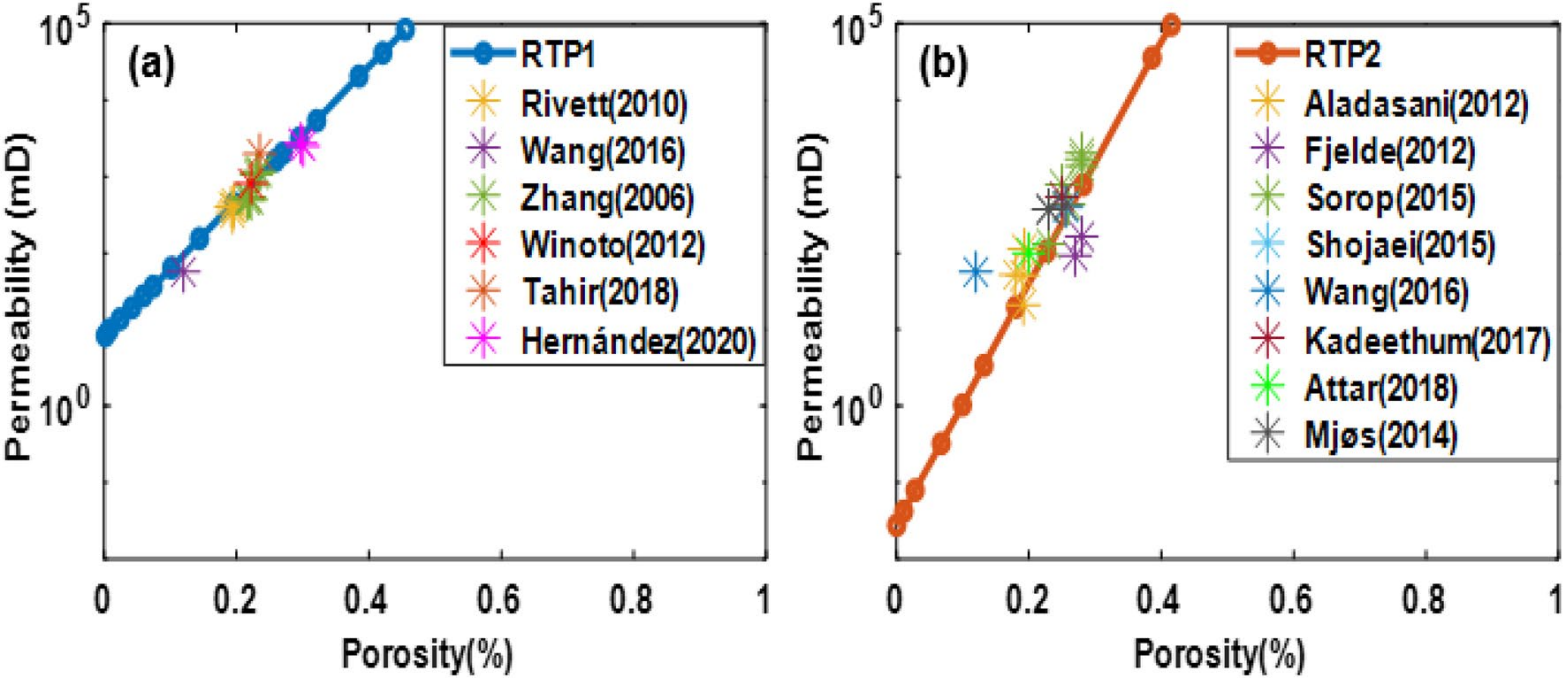
The distribution of porosity and permeability for RTP1 (**a**) and RTP2 (**b**).

Moreover, the distribution of porosity and permeability in the investigated studies are compared to ours as in Fig. 2:

After categorizing the conducted studies into two different rock types, the gathered data from these studies is used to determine the HM parameters of both rock types, which will be discussed in the following sections.

### Coupling reservoir simulator and PSO algorithm to determine model parameters

If the relative permeability and capillary pressure parameters are not reported directly, the matching parameters must be obtained by coupling the reservoir simulator and the PSO algorithm (AHM). The Coupling procedure to obtain the optimized model parameters is described in 9 steps:

1. Preparing the initial swarm; n values (n = 50 in this study) are given to each parameter of the swarm.
2. Calculating relative permeability and capillary pressure values for each parameter. Corey’s model is used to calculate water and oil relative permeability:
  1 $${{\text{k}}}_{{\text{rw}}}={{\text{k}}}_{{\text{rw}}}^{0}\times {({{\text{S}}}_{{\text{wn}}})}^{{{\text{n}}}_{{\text{w}}}}$$
  2 $${{\text{k}}}_{{\text{ro}}}={{\text{k}}}_{{\text{ro}}}^{0}\times {(1-{{\text{S}}}_{{\text{wn}}})}^{{{\text{n}}}_{{\text{o}}}}$$

In the above equations,${{\text{k}}}_{{\text{rw}}/{\text{o}}}^{0}$ water/oil endpoint relative permeability and ${{\text{n}}}_{{\text{o}}/{\text{w}}}$ are Corey exponent parameters, which would be obtained by AHM.

3 $${{\text{J}}}_{\left({{\text{S}}}_{{\text{w}}}\right)}^{{\text{m}}}=\frac{{{\text{a}}}_{1}}{1+{{\text{k}}}_{1}{{\text{S}}}_{{\text{nw}}}}-\frac{{{\text{a}}}_{2}}{1+{{\text{k}}}_{2}{(1-{\text{S}}}_{{\text{nw}}})}+{{\text{b}}}_{1}$$

4 $$- 1 < {\text{J}}^{{\text{m}}} { } < 1.{\text{ P}}_{{{\text{cow}}}} \left( {{\text{S}}_{{{\text{nw}}}} } \right) = {\text{P}}_{{{\text{cowmax}}}} {\text{J}}_{{{\text{S}}_{{{\text{nw}}}} }}$$

In Eq. 3,${{\text{a}}}_{1}$, ${{\text{k}}}_{1}$,${{\text{a}}}_{2}$, ${{\text{k}}}_{2}$, and ${{\text{b}}}_{1}$ are correlation parameters. ${{\text{P}}}_{{\text{cowmax}}}$ in Eq. 4 presents maximum capillary pressure. If the values of relative permeability and capillary pressure are not reported in the investigated studies, the constant parameters of the capillary pressure, along with Corey’s equation, would be determined by AHM.

3. Creating include file.
4. Run MATLAB coupled with reservoir simulator.
5. Post Processing: The exclude file is an Excel file containing the results of oil recovery and pressure drop; other results would be omitted. In HM, rock properties (permeability and porosity), fluid properties (viscosity, compressibility, density, and API), and rock-fluid properties (${{\text{k}}}_{{\text{r}}}$ and ${{\text{P}}}_{{\text{C}}}$) are considered as inputs. If the model result does not match the experimental data, then model parameters are changed using the HM algorithm (PSO), and it is rerun**.**
6. The final purpose is to minimize the difference between the model and experimental results, evaluating the objective function as Eq. 5:
  5 $${\text{ERROR}}=\sqrt{{\left({(\left\{{\text{RF}}\right\}.\left\{\Delta {\text{P}}\right\})}_{{\text{Sim}}}-{(\left\{{\text{RF}}\right\}.\left\{\Delta {\text{P}}\right\})}_{{\text{Exp}}}\right)}^{2}}$$

The model can be used to predict reservoir performance when the difference between predicted ${(\left\{{\text{RF}}\right\}.\left\{\Delta {\text{P}}\right\})}_{{\text{Sim}}}$ and measured values ${(\left\{{\text{RF}}\right\}.\left\{\Delta {\text{P}}\right\})}_{{\text{Exp}}}$ is minimized^4^.

7. Optimizing the errors, Pbest (the best solution of particle i) and Gbest (the best solution of all particles) are calculated.
8. Creating a new swarm based on Pbest and Gbest.
9. Repeating 2–8 steps to reach the lowest error or convergence.

**Figure 3:**
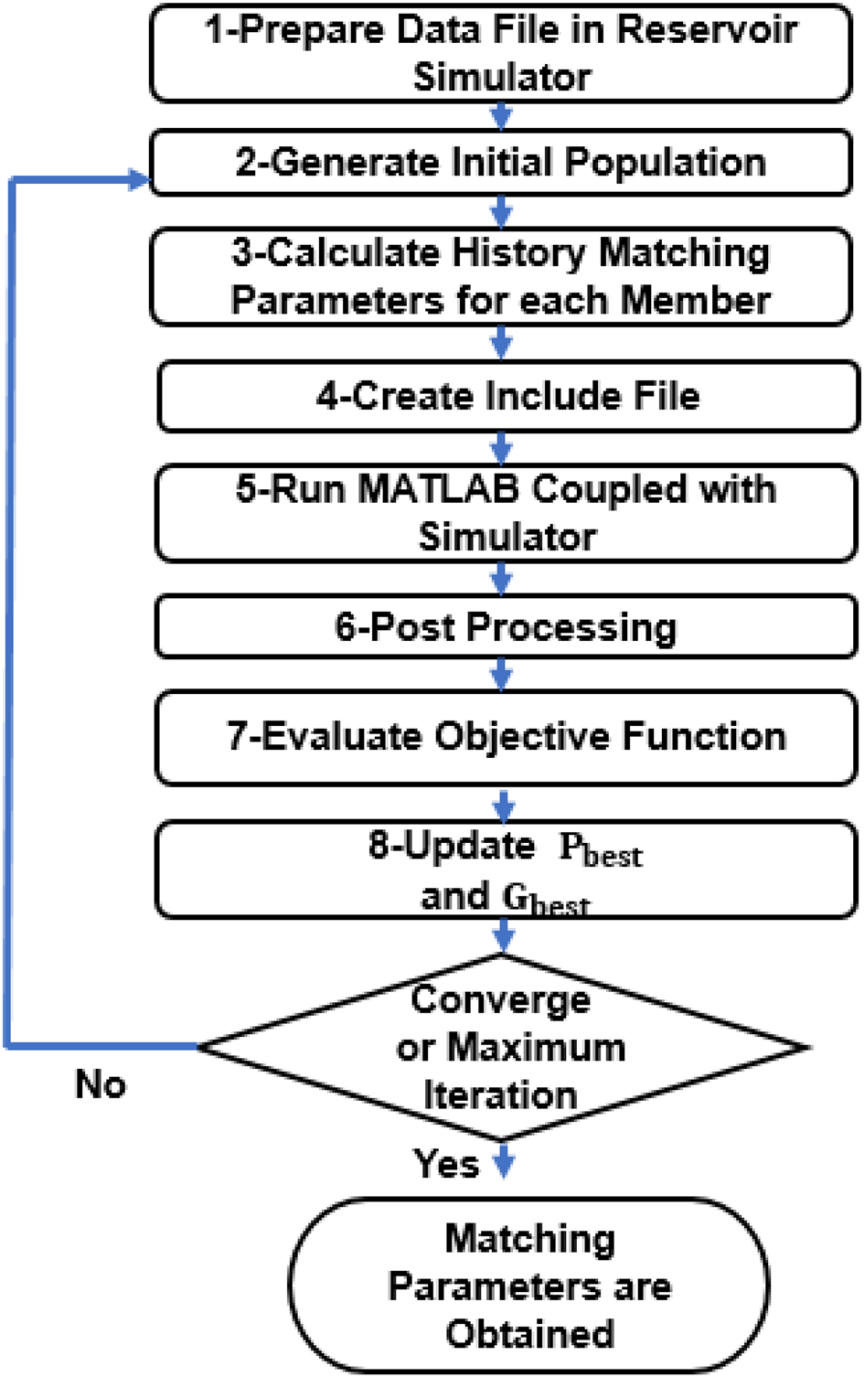
The overall fellow chart of coupling the simulator and PSO to obtain optimized model parameters.

The overall fellow chart of coupling the simulator and PSO to obtain optimized model parameters is shown in Fig. 3:

By coupling the simulator and PSO algorithm, experimental results of recovery factor and pressure drop are matched with simulated results, and relative permeability and capillary pressure curves are determined as matching parameters. The summarized variation of optimized parameters for RTP1 is reported in Table 4:

**Table 4:** The summarized range of optimized relative permeability parameters for RTP1.

Refs.	${{\text{n}}}_{{\text{w}}}$(High Sal)	${{\text{n}}}_{{\text{o}}}$(High Sal)	${{\text{k}}}_{{\text{ro}}}^{{\text{end}}}$(High Sal)	${{\text{k}}}_{{\text{rw}}}^{{\text{end}}}$(High Sal)	${{\text{S}}}_{{\text{ro}}}$(High Sal)
	${{\text{n}}}_{{\text{w}}}$(Low Sal)	${{\text{n}}}_{{\text{o}}}$(Low Sal)	${{\text{k}}}_{{\text{ro}}}^{{\text{end}}}$(Low Sal)	${{\text{k}}}_{{\text{rw}}}^{{\text{end}}}$(Low Sal)	${{\text{S}}}_{{\text{ro}}}$(Low Sal)
High:38,000 Low:386 Zhang^41^	1.69	2.60	0.82	0.3	0.31
	2.33	2.48	0.88	0.14	0.21
High:30,000 Low:1140 Rivett^42^	2.15	1.37	0.28	0.14	0.3
	3.2	1.21	0.38	0.05	0.2
High:35,000 Low:17,000 Winoto^43^	1.34	2.19	0.42	0.35	0.33
	1.49	2.047	0.55	0.23	0.2
High:57,000 Low:2800 Wang^44^	1.33	2.8	0.68	0.56	0.17
	2.07	1.96	0.84	0.13	0.11
High:40,000 Low:4000 Tahir^3^	1.25	1.10	0.68	0.33	0.24
	1.37	1.02	0.76	0.16	0.18
High:36,000 Low:1035 Hernández^45^	1.55	1.7	0.9	0.53	0.25
	2.08	1.61	0.99	0.28	0.17

The summarized variation of optimized parameters for RTP2 is reported in Table 5:

**Table 5:** The summarized range of optimized relative permeability parameters for RTP2.

Ref	${\mathbf{n}}_{\mathbf{w}}$(High Sal)	${\mathbf{n}}_{\mathbf{o}}$(High Sal)	${\mathbf{k}}_{\mathbf{r}\mathbf{o}}^{\mathbf{e}\mathbf{n}\mathbf{d}}$(High Sal)	${\mathbf{k}}_{\mathbf{r}\mathbf{w}}^{\mathbf{e}\mathbf{n}\mathbf{d}}$(High Sal)	${\mathbf{S}}_{\mathbf{r}\mathbf{o}}$(High Sal)
	${\mathbf{n}}_{\mathbf{w}}$(Low Sal)	${\mathbf{n}}_{\mathbf{o}}$(Low Sal)	${\mathbf{k}}_{\mathbf{r}\mathbf{o}}^{\mathbf{e}\mathbf{n}\mathbf{d}}$(Low Sal)	${\mathbf{k}}_{\mathbf{r}\mathbf{w}}^{\mathbf{e}\mathbf{n}\mathbf{d}}$(Low Sal)	${\mathbf{S}}_{\mathbf{r}\mathbf{o}}$(Low Sal)
High:38,000 Low:1000 Aladasani^47^	2.16	2.12	0.61	0.15	0.2
	2.97	1.6	0.8	0.09	0.03
High:33,000 Low:1000 Kozaki^46^	1.6	4.19	0.57	0.026	0.38
	2.74	4.11	0.65	0.013	0.35
High:105,000 Low:1000 Fjelde^48^	2.7	2.5	0.8	0.2	0.3
	3.6	4	0.92	0.04	0.19
High:36,000 Low:3000 Mjøs^49^	1.63	1.95	0.62	0.16	0.31
	2.45	1.44	0.82	0.12	0.21
High:35,000 Low:2500 Shojaei^50^	1.8	1.9	0.98	0.06	0.22
	2.6	1.4	0.98	0.04	0.12
High:26,000 Low:1500 Sorop^1^	1.16	2.74	0.75	0.59	0.18
	2.51	2.34	0.76	0.39	0.11
High:20,000 Low:700 Kadeethum^51^	2.8	5.84	0.97	0.28	0.34
	3.7	1.39	0.99	0.18	0.29
High:30,000 Low:1000 Attar^52^	2.1	2.8	0.89	0.38	0.35
	3.7	2	0.99	0.28	0.27

Similarly, for capillary pressure, matching parameters are also obtained in each study for both rock types. HM parameters reported in Tables 4 and 5 are computed with approximately less than 5% error and a running time of 2.5 h. High salinity relative permeability curves for studies similar to RTP1 and RTP2 were compared with those of our cases.

### Proposed correlation for relative permeability parameters (machine learning technique)

Having the matching parameters in two different salinities (high and low), in other low-salinity conditions the relative permeability and capillary pressure parameters were estimated through linear interpolation, and finally by using multi-linear regression, the correlations were developed.

For RTP1 (no clay), the correlations for relative permeability and capillary pressure parameters are proposed using multiple-linear regression as Eqs. 6–10 for every desired low-salinity condition (1000–10000 ppm). The experimental conditions for developing correlations include the porosity range of 0.15–0.4, permeability range of 0.01–100 Darcy, mixed wet wettability, and clay-free formation.

6 $$\begin{aligned} {\mathbf{n}}_{{{\mathbf{w}}\left( {{\mathbf{LSW}}} \right)}} & = - {\text{2}}.{\text{32}} + \left[ {\left( {{\text{2}}.{\text{58E}} - 0{\text{5}}} \right) \times \left( {{\text{HSW}}\,{\text{Concentration}}} \right)} \right] \\ &\quad+ [\left( {{\text{2}}.{\text{19}}} \right) \times ({\text{n}}_{{{\text{w}}\left( {{\text{HSW}}} \right)}} )\left] - \right[\left( {{\text{8}}.{\text{2E}} - 0{\text{6}}} \right) \times \left( {{\text{LSW}}\,{\text{Concentration}}} \right)] \\ {\text{R}}^{2} & = 0 \cdot 98.~~{\text{MSE}} = 1{\text{E}} - 04 \\ \end{aligned}$$

7 $$\begin{aligned} {\mathbf{n}}_{{{\mathbf{o}}\left( {{\mathbf{LSW}}} \right)}} & = {1}.{14} - \left[ {({2}.{\text{9E}} - 0{5}) \times ({\text{HSW}}\,{\text{Concentration}})] + } \right[(0.{895}) \times ({\text{n}}_{{{\text{o}}\left( {{\text{HSW}}} \right)}} )] + \left[ {\left( {{2}.{\text{4E}} - 0{6}} \right) \, \times \, \left( {{\text{LSW}}\,{\text{Concentration}}} \right)} \right] \\ {\text{R}}^{2} & = 0 \cdot 98. {\text{MSE}} = 6{\text{E}} - 04 \\ \end{aligned}$$

8 $$\begin{aligned} {\mathbf{kr}}_{{{\mathbf{w}}\left( {{\mathbf{LSW}}} \right)}}^{{{\mathbf{end}}}} & = 0.{\text{279}} - \left[ {\left( {{\text{8}}.{\text{1E}} - 0{\text{6}}} \right) \times \left( {{\text{HSW}}\,{\text{Concentration}}} \right)} \right]\\ &\quad + [\left( {0.{\text{545}}} \right){\text{ }} \times ({\text{kr}}_{{{\text{w}}\left( {{\text{HSW}}} \right)}}^{{{\text{end}}}} )\left] + \right[\left( {{\text{6E}} - 0{\text{6}}} \right) \times \left( {{\text{LSW}}\,{\text{Concentration}}} \right)] \\ {\text{R}}^{2} & = 0 \cdot 98.~~{\text{MSE}} = 7{\text{E}} - 05 \\ \end{aligned}$$

9 $$\begin{aligned} {\mathbf{kr}}_{{{\mathbf{o}}\left( {{\mathbf{LSW}}} \right)}}^{{{\mathbf{end}}}} & = 0.{149} + \left[ {\left( {{2}.{\text{39E}} - 0{6}} \right) \times \left( {{\text{HSW}}\,{\text{Concentration}}} \right)} \right] \\ &\quad+ [\left( {0.{81}} \right) \times ({\text{kr}}_{{{\text{o}}\left( {{\text{HSW}}} \right)}}^{{{\text{end}}}} )\left] - \right[\left( {{2}.{\text{9E}} - 0{6}} \right) \times ({\text{LSW}}\,{\text{Concentration}})] \\ {\text{R}}^{2} & = 0 \cdot 97. {\text{MSE}} = 5{\text{E}} - 05 \\ \end{aligned}$$

10 $$\begin{aligned} {\mathbf{S}}_{{{\mathbf{or}}\left( {{\mathbf{LSW}}} \right)}} & = 0.0{97} - \left[ {({1}.{\text{3E}} - 0{6}) \times ({\text{HSW}}\,{\text{Concentration}})] + } \right[\left( {0.{46}} \right) \times ({\text{S}}_{{{\text{or}}\left( {{\text{HSW}}} \right)}} )] \\ &\quad+ [\left( {{3}.{\text{44E}} - 0{6}} \right) \times ({\text{LSW}}\,{\text{Concentration}})] \\ {\text{R}}^{2} & = 0 \cdot 95. {\text{MSE}} = 8{\text{E}} - 04 \\ \end{aligned}$$

11 $$\begin{aligned} {\mathbf{P}}_{{{\mathbf{Cmax}}\left( {{\mathbf{LSW}}} \right)}} & = - {13}.{44} + \left[ {\left( {{1}.{\text{2E}} - 0{4}} \right) \times \left( {{\text{HSW}}\,{\text{Concentration}}} \right)} \right] \\ &\quad+ [\left( {0.{93}} \right) \times ({\text{P}}_{{{\text{Cmax}}\left( {{\text{HSW}}} \right)}} )\left] + \right[\left( {{7}.{\text{58E}} - 0{4}} \right) \times \left( {{\text{LSW}}\,{\text{Concentration}}} \right)] \\ {\text{R}}^{2} & = 0 \cdot 93. {\text{MSE}} = 1 \cdot 2{\text{E}} - 02 \\ \end{aligned}$$

12 $$\begin{aligned} {\mathbf{a}}_{{1\left( {{\mathbf{LSW}}} \right)}} & = 0.{\text{314}} - \left[ {({\text{8}}.{\text{51E}} - 0{\text{6}}) \times ({\text{HSW}}\,{\text{Concentration}})] + } \right[({\text{1}}.{\text{35}}) \times ({\text{a}}_{{1\left( {{\text{HSW}}} \right)}} )] - \left[ {\left( {{\text{2}}.{\text{6E}} - 0{\text{5}}} \right) \times \left( {{\text{LSW}}\,{\text{Concentration}}} \right)} \right] \\ {\text{R}}^{2} & = 0 \cdot 90.~~{\text{MSE}} = 4 \cdot 3{\text{E}} - 01 \\ \end{aligned}$$

13 $$\begin{aligned} {\mathbf{a}}_{{2\left( {{\mathbf{LSW}}} \right)}} & = 0.0{34} + \left[ {\left( {{2}.{\text{3E}} - 0{7}} \right) \times \left( {{\text{HSW}}\,{\text{Concentration}}} \right)} \right] + [\left( {{1}.{22}} \right) \times ({\text{a}}_{{2\left( {{\text{HSW}}} \right)}} )\left] - \right[\left( {{1}.{\text{6E}} - 0{5}} \right) \times \left( {{\text{LSW}}\,{\text{Concentration}}} \right)] \\ {\text{R}}^{2} & = 0 \cdot 99.{\text{ MSE}} = 6 \cdot 7{\text{E}} - 05 \\ \end{aligned}$$

14 $$\begin{aligned} {\mathbf{k}}_{{2\left( {{\mathbf{LSW}}} \right)}} & = - {68}.{44} + \left[ {({1}.{\text{3E}} - 0{3}) \times ({\text{HSW}}\,{\text{Concentration}})] + } \right[({1}.{13}) \times ({\text{k}}_{{2\left( {{\text{HSW}}} \right)}} )] + [\left( {{\text{8E}} - 0{4}} \right) \times ({\text{LSW}}\,{\text{Concentration}})] \\ {\text{R}}^{2} & = 0 \cdot 97. {\text{MSE}} = 3 \cdot 4{\text{E}} - 04 \\ \end{aligned}$$

In the above equations, (HSW Concentration) is the concentration of high-salinity water, and (LSW Concentration) is the concentration of low-salinity water. The subscript of each of the coefficients indicates high and low salinity conditions.

For RTP2 (clayey formation), the proposed correlations are as Eqs. 11–15 for every desired low-salinity case. The experimental conditions for developing correlations include the porosity range of 0.1–0.35, permeability range of 0.00001–100 Darcy, weakly water wet wettability, and clayey formation. Moreover, these correlations are used for the salinity range between 1000 and 10,000 ppm:

15 $$\begin{aligned} {\mathbf{n}}_{{{\mathbf{w}}\left( {{\mathbf{LSW}}} \right)}} & = {2}.0{63} - \left[ {\left( {{\text{1E}} - 0{5}} \right) \times \left( {{\text{HSW}}\,{\text{Concentration}}} \right)} \right] + [\left( {0.{6}0{1}} \right) \times ({\text{n}}_{{{\text{w}}\left( {{\text{HSW}}} \right)}} )\left] - \right[\left( {{\text{2E}} - 0{5}} \right) \times \left( {{\text{LSW}}\,{\text{Concentration}}} \right)] \\ {\text{R}}^{2} & = 0 \cdot 94. {\text{MSE}} = 9 \cdot 09{\text{E}} - 05 \\ \end{aligned}$$

16 $$\begin{aligned} {\mathbf{n}}_{{{\mathbf{o}}\left( {{\mathbf{LSW}}} \right)}} & = - 0.{611} - \left[ {\left( {{\text{8E}} - 0{6}} \right) \times \left( {{\text{HSW}}\,{\text{Concentration}}} \right)} \right] \\ &\quad+ [\left( {{1}.{17}} \right) \times ({\text{n}}_{{{\text{o}}\left( {{\text{HSW}}} \right)}} )\left] + \right[\left( {{1}.{\text{62E}} - 0{5}} \right) \times \left( {{\text{LSW}}\,{\text{Concentration}}} \right)] \\ {\text{R}}^{2} & = 0 \cdot 98. {\text{MSE}} = 1 \cdot 3{\text{E}} - 05 \\ \end{aligned}$$

17 $$\begin{aligned} {\mathbf{kr}}_{{{\mathbf{w}}\left( {{\mathbf{LSW}}} \right)}}^{{{\mathbf{end}}}} & = 0.0{\text{25}} - \left[ {\left( {{\text{2}}.{\text{2E}} - 0{\text{6}}} \right) \times \left( {{\text{HSW}}\,{\text{Concentration}}} \right)} \right] \\ &\quad+ [\left( {0.{\text{778}}} \right) \times ({\text{kr}}_{{{\text{w}}\left( {{\text{HSW}}} \right)}}^{{{\text{end}}}} )\left] + \right[\left( {{\text{7}}.{\text{73E}} - 0{\text{6}}} \right) \times \left( {{\text{LSW}}\,{\text{Concentration}}} \right)] \\ {\text{R}}^{2} & = 0 \cdot 97.~~{\text{MSE}} = 3 \cdot 37{\text{E}} - 06 \\ \end{aligned}$$

18 $$\begin{aligned} {\mathbf{kr}}_{{{\mathbf{o}}\left( {{\mathbf{LSW}}} \right)}}^{{{\mathbf{end}}}} & = - 0.{89} + \left[ {\left( {{1}.{\text{99E}} - 0{5}} \right) \times \left( {{\text{HSW}}\,{\text{Concentration}}} \right)} \right] \\ &\quad+ [\left( {{1}.{57}} \right) \, \times \, ({\text{kr}}_{{{\text{o}}\left( {{\text{HSW}}} \right)}}^{{{\text{end}}}} )\left] - \right[\left( {{3}.{\text{9E}} - 0{6}} \right) \, \times \, ({\text{LSW}}\,{\text{Concentration}})] \\ {\text{R}}^{2} & = 0 \cdot 94. {\text{MSE}} = 1 \cdot 1{\text{E}} - 05 \\ \end{aligned}$$

19 $$\begin{aligned} {\mathbf{S}}_{{{\mathbf{or}}\left( {{\mathbf{LSW}}} \right)}} & = 0.0{97} - \left[ {({9}.{\text{6E}} - 0{6}) \times ({\text{HSW}}\,{\text{Concentration}})] + } \right[({1}.{45}) \times ({\text{S}}_{{{\text{or}}\left( {{\text{HSW}}} \right)}} )] \\ &\quad+ [\left( {{3}.{\text{17E}} - 0{6}} \right) \, \times \, ({\text{LSW}}\,{\text{Concentration}})] \\ {\text{R}}^{2} & = 0 \cdot 99. {\text{MSE}} = 3 \cdot 1{\text{E}} - 05 \\ \end{aligned}$$

20 $$\begin{aligned} {\mathbf{P}}_{{{\mathbf{Cmax}}\left( {{\mathbf{LSW}}} \right)}} & = - {24}.{83} + \left[ {\left( {{6}.{\text{95E}} - 0{4}} \right) \times \left( {{\text{HSW}}\,{\text{Concentration}}} \right)} \right] \\ &\quad+ [\left( {0.{55}} \right) \times ({\text{P}}_{{{\text{Cmax}}\left( {{\text{HSW}}} \right)}} )\left] + \right[\left( {{8}.{\text{98E}} - 0{4}} \right) \times \left( {{\text{LSW}}\,{\text{Concentration}}} \right)] \\ {\text{R}}^{2} & = 0 \cdot 92. {\text{MSE}} = 2 \cdot 3{\text{E}} - 02 \\ \end{aligned}$$

21 $$\begin{aligned} {\mathbf{a}}_{{1\left( {{\mathbf{LSW}}} \right)}} & = {1}.{4} - \left[ {({3}.{\text{6E}} - 0{5}) \times ({\text{HSW}}\,{\text{Concentration}})] + } \right[({1}.{17}) \times ({\text{a}}_{{1\left( {{\text{HSW}}} \right)}} )] - \left[ {\left( {{2}.{\text{5E}} - 0{5}} \right) \times \left( {{\text{LSW}}\,{\text{Concentration}}} \right)} \right] \\ {\text{R}}^{2} & = 0 \cdot 97. {\text{MSE}} = 6 \cdot 5{\text{E}} - 04 \\ \end{aligned}$$

22 $$\begin{aligned} {\mathbf{a}}_{{2\left( {{\mathbf{LSW}}} \right)}} & = - 0.{37} + \left[ {({4}.{\text{5E}} - 0{6}) \times ({\text{HSW}}\,{\text{Concentration}})] + } \right[({1}.{7}) \times ({\text{a}}_{{2\left( {{\text{HSW}}} \right)}} )] - \left[ {\left( {{\text{3E}} - 0{5}} \right) \times \left( {{\text{LSW}}\,{\text{Concentration}}} \right)} \right] \\ {\text{R}}^{2} & = 0 \cdot 90. {\text{MSE}} = 7 \cdot 2{\text{E}} - 02 \\ \end{aligned}$$

23 $$\begin{aligned} {\mathbf{k}}_{{2\left( {{\mathbf{LSW}}} \right)}} & = - {6}.{2} + \left[ {({2}.{\text{8E}} - 0{7}) \times ({\text{HSW}}\,{\text{Concentration}})] + } \right[({1}.0{1}) \times ({\text{k}}_{{2\left( {{\text{HSW}}} \right)}} )] + [\left( {{4}.{\text{19E}} - 0{4}} \right) \times ({\text{LSW}}\,{\text{Concentration}})] \\ {\text{R}}^{2} & = 0 \cdot 99. {\text{MSE}} = 7 \cdot 5{\text{E}} - 05 \\ \end{aligned}$$

Since few similar studies were available, the results of Rivett for RTP1^42^, and Shojaei for RTP2^50^ were used to validate the developed correlations and is presented in Table 6:

**Table 6:** Validation of the proposed correlations for RTP1 and RTP2 against reported experimental data.

Refs.	${\mathbf{n}}_{\mathbf{w}}$(Low Sal)		${\mathbf{n}}_{\mathbf{o}}$(Low Sal)		${\mathbf{k}}_{\mathbf{r}\mathbf{w}}^{\mathbf{e}\mathbf{n}\mathbf{d}}$(Low Sal)		${\mathbf{k}}_{\mathbf{r}\mathbf{o}}^{\mathbf{e}\mathbf{n}\mathbf{d}}$(Low Sal)		${\mathbf{S}}_{\mathbf{o}\mathbf{r}}$(Low Sal)	
	Correlation	Exp	Correlation	Exp	Correlation	Exp	Correlation	Exp	Correlation	Exp
Rivet^42^ (RTP1)	3.35	3.2	1.19	1.21	0.06	0.05	0.44	0.38	0.19	0.2
Shojaei^50^ (RTP2)	2.62	2.6	1.37	1.4	0.03	0.04	0.99	0.99	0.121	0.12

Although the data from these two studies were not used to develop oil and water relative permeability correlations, a suitable match was observed (R^2^ = 0.95, MSE = 1.2E-04 for RTP1 and R^2^ = 0.97, MSE = 8E-05 for RTP2). Therefore, it is possible to use these proposed correlations to determine the relative permeability and capillary pressure functions at every desired low-salinity condition, within the scope of the mentioned conditions with high accuracy.

### Averaging relative permeability and capillary pressure curves

Among the rock properties, capillary pressure and relative permeability are more challenging, as are functions of fluids’ saturation. TEM function is calculated by having relative permeability, permeability, porosity, and viscosity. Equation 24 is similar to the J function and is extensively used to scale the capillary pressure curve^27^. The average relative permeability should be determined for each rock type as a function of the normalized saturation. The weighted relative permeability approach (Eq. 25) determines the average relative permeability curve for RTP1 and RTP2. The obtained pseudo relative permeability curve in this method varies between zero and one^27,36^.

In Eq. 17, ${{{\text{kr}}}_{{\text{a}}}}_{({\text{Ave}})}$ is the average phase relative permeability for each rock type, ${{\text{T}}}_{{\text{x}}}$ is the phase transmissibility, ${\upmu }_{{\text{a}}}$ is the phase viscosity, $\mathrm{\varphi }$ is the rock’s porosity, and ${\text{k}}$ is the rock’s absolute permeability.

24 $${{\text{TEM}}}_{{\text{a}}}=\frac{{{\text{kk}}}_{{\text{ra}}}}{\mathrm{\varphi }{\upmu }_{{\text{a}}}}$$

25 $${{{\text{kr}}}_{{\text{a}}}}_{({\text{Ave}})}=\frac{\sum_{{\text{i}}=1}^{{\text{n}}}{[{\text{T}}}_{{\text{x}}}{{\text{k}}}_{{\text{ra}}}{]}_{{\text{i}}}}{\sum_{{\text{i}}=1}^{{\text{n}}}{[{\text{T}}}_{{\text{X}}}{]}_{{\text{i}}}}=\frac{\sum_{{\text{i}}=1}^{{\text{n}}}[{{\text{TEM}}}_{{\text{a}}}{]}_{{\text{i}}}}{\sum_{{\text{i}}=1}^{{\text{n}}}[\frac{{\text{k}}}{\mathrm{\varphi }{\upmu }_{{\text{a}}}}{]}_{{\text{i}}}}=\frac{\sum_{{\text{i}}=1}^{{\text{n}}}[\frac{{{\text{kk}}}_{{\text{ra}}}}{\mathrm{\varphi }{\upmu }_{{\text{a}}}}{]}_{{\text{i}}}}{\sum_{{\text{i}}=1}^{{\text{n}}}[\frac{{\text{k}}}{\mathrm{\varphi }{\upmu }_{{\text{a}}}}{]}_{{\text{i}}}}$$

“The Leverett J” is a dimensionless number relating capillary pressure to rock and fluid properties such as porosity, interfacial tension, and mean pore radius. It is the most common method used for averaging capillary pressure curves and can be calculated using Eq. 26^6^:

26 $${{\text{J}}}_{({\text{Sw}})}=0\cdot 2166\times {\text{Pc}}\times \frac{\sqrt{\frac{{\text{k}}}{\mathrm{\varphi }}}}{\upsigma \times \mathrm{cos\theta }}$$

Pc is capillary pressure (psi), σ is the interfacial tension (0.27 dynes/cm), θ is the fluid’s contact angle (θ = 0), and J is a dimensionless value. To estimate the average capillary pressure using J-function, the following steps are required:

1. Equation 26 converts the capillary pressure values to the J-function.
2. Calculation of the average J-function for a block by using the pore volume averaging is as Eq. 27:
  27 $${{\text{J}}}_{{\text{ave}}}=\frac{\sum_{{\text{i}}=1}^{{\text{n}}}{{\text{J}}}_{{\text{i}}}{{\text{V}}}_{{\text{i}}}{\mathrm{\varphi }}_{{\text{i}}}}{\sum_{{\text{i}}=1}^{{\text{n}}}{{\text{V}}}_{{\text{i}}}{\mathrm{\varphi }}_{{\text{i}}}}$$

${{\text{V}}}_{{\text{i}}}$ and ${\mathrm{\varphi }}_{{\text{i}}}$ stand for each rock sample’s bulk volume and porosity, respectively.

3. The average capillary pressure is finally calculated (Eq. 28) by having the average J-function values (Eq. 27).
  28 $${{\text{P}}}_{\mathrm{c }({\text{ave}})}=\frac{{{\text{J}}}_{{\text{ave}}}\times\updelta \times }{0\cdot 2166}\sqrt{\frac{{{\text{K}}}_{{\text{ave}}}}{{\mathrm{\varphi }}_{{\text{ave}}}}}$$

## Results

**Figure 4:**
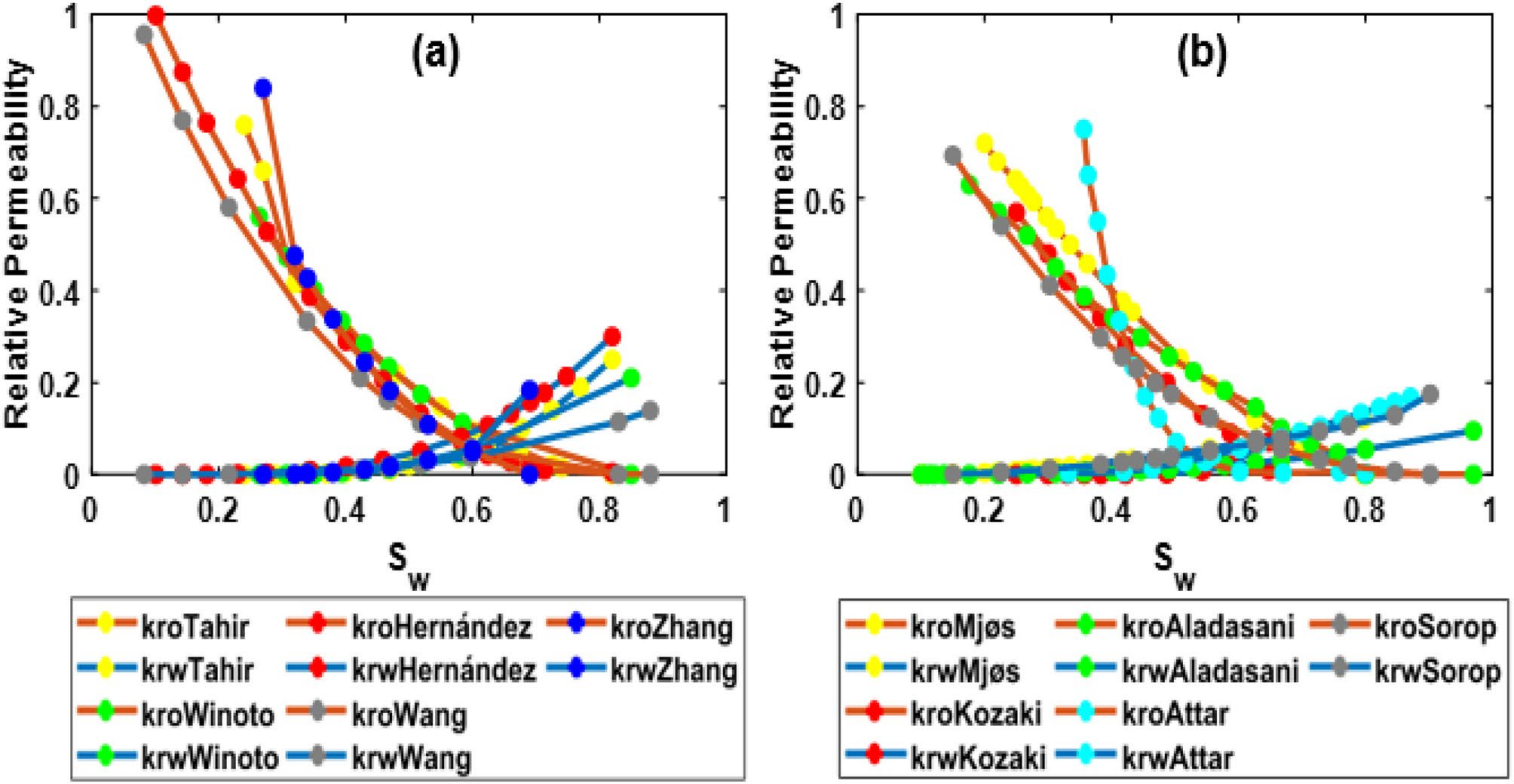
The low-salinity (4000 ppm) relative permeability curves from all studies of RTP1 (**a**) and RTP2 (**b**).

The HSW for our case is 42,000 ppm (sea water). Based on the literature review^3,46,51,53–59^, approximately ten times the dilution of HSW could be considered as the optimized salinity. All relative permeability and capillary pressure curves obtained from studies (RTP1 and RTP2) are calculated to a low salinity of 4000 ppm (based on developed correlations). The low-salinity (4000 ppm) relative permeability curves from all studies of RTP1 and RTP2 are shown in Fig. 4a,b:

### Averaged relative permeability and capillary pressure curve for a low salinity case

**Figure 5:**
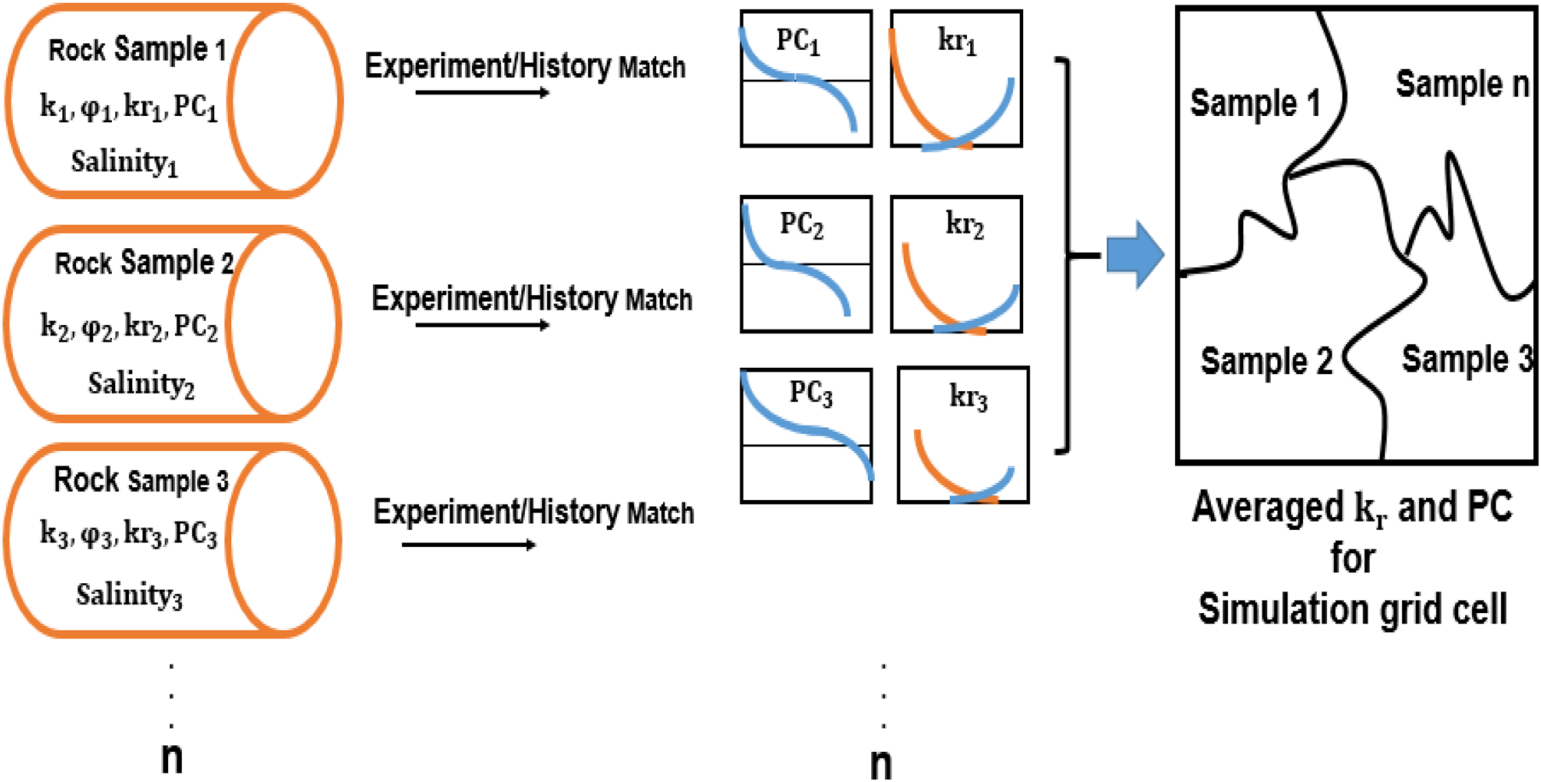
Averaging process of the relative permeability and capillary pressure curves.

As mentioned, the size of grid cells in the simulator for large-scale applications is much larger than laboratory core plugs where rock properties are measured. Thus, averaging techniques should be used to obtain simulation block properties^6^ (Fig. 5).

**Figure 6:**
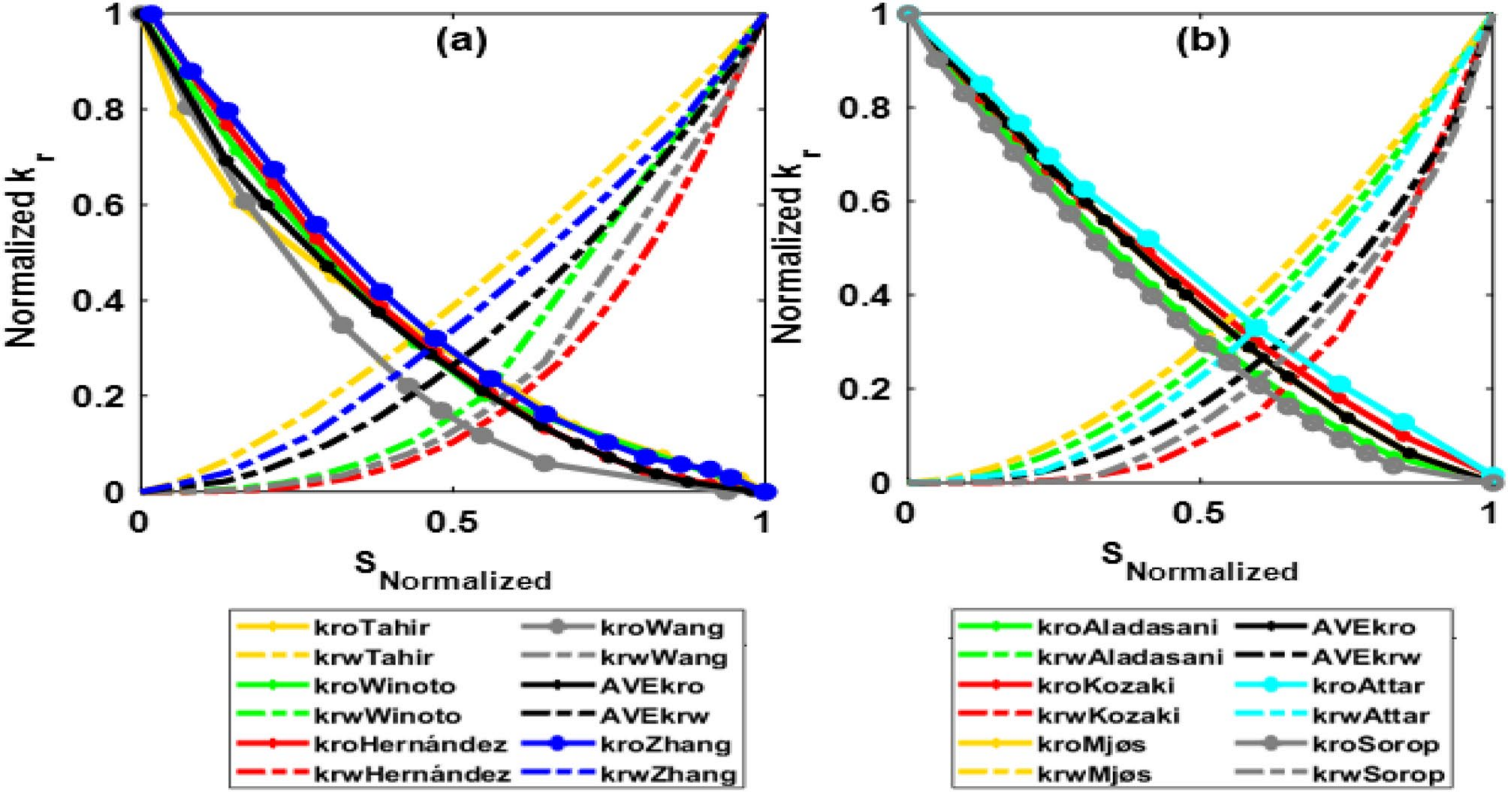
Averaging relative permeability model parameters in normalized form for RTP1 (**a**) and RTP2 (**b**).

**Figure 7:**
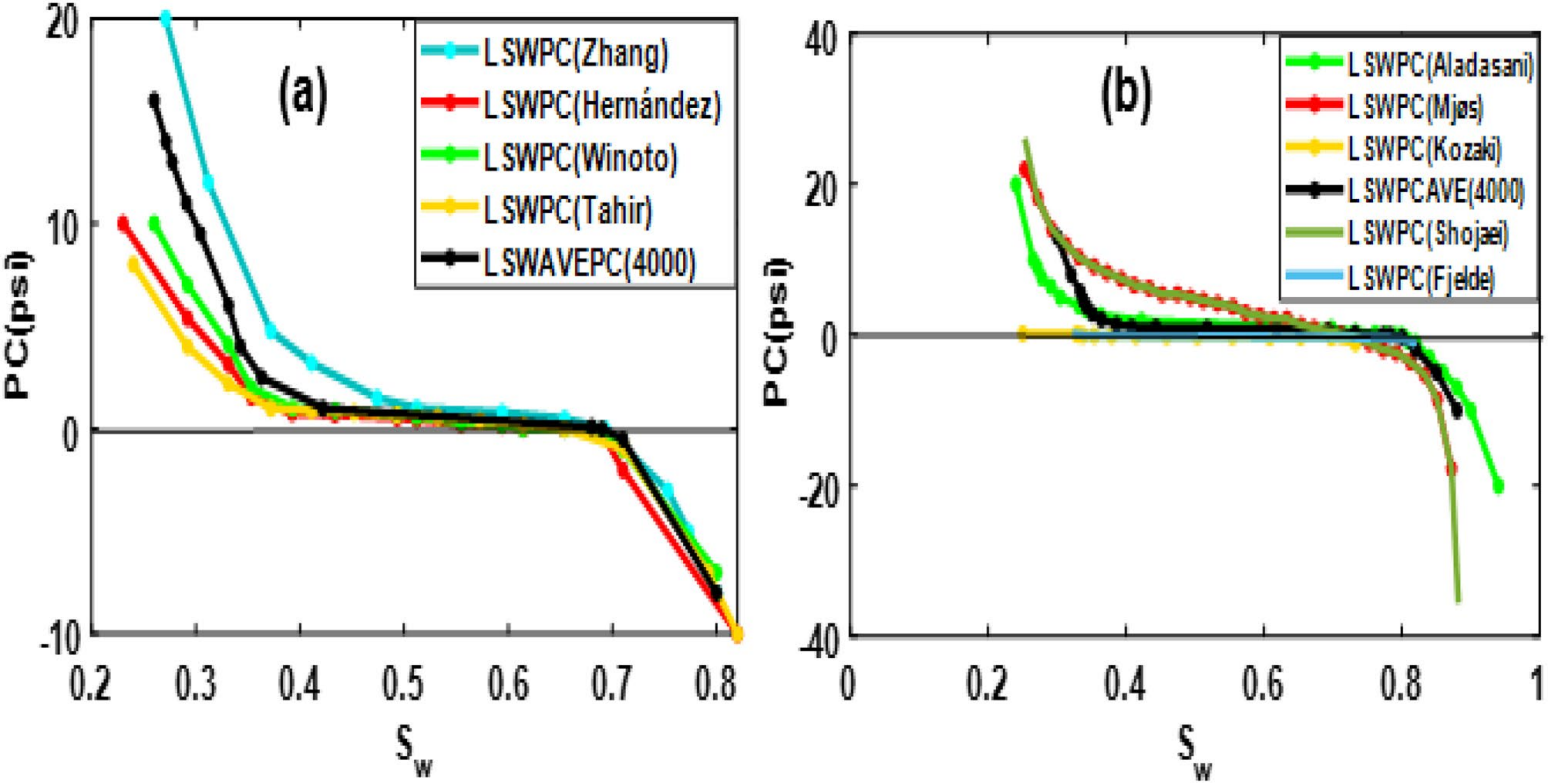
Averaging model parameters and converting to averaged Pc for RTP1 (**a**) and RTP2 (**b**).

The obtained pseudo relative permeability curves for RTP1 and RTP2 using the weighted relative permeability approach for a low-salinity case of 4000 ppm are shown in Fig. 6:

The average capillary pressure curves for RTP1 and RTP2 for a low-salinity case of 4000 ppm are also illustrated in Fig. 7a,b:

Finally, a single curve for capillary pressure and relative permeability would be assigned to each grid cell within a rock type.

### Investigating the effect of salinity and clay content on the obtained averaged curves

**Figure 8:**
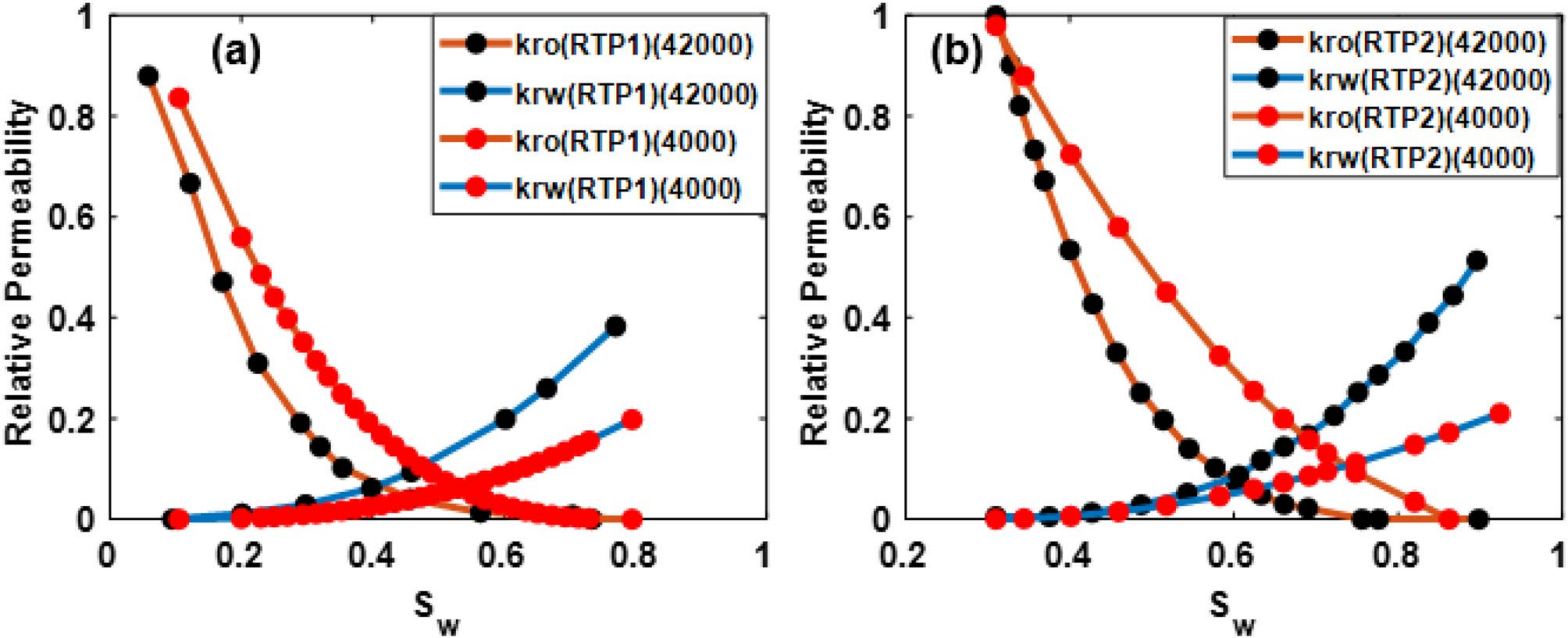
Comparison of HSW (42,000) and LSW (4000 ppm) water/oil relative permeability curves for RTP1 (**a**) and RTP2 (**b**).

**Figure 9:**
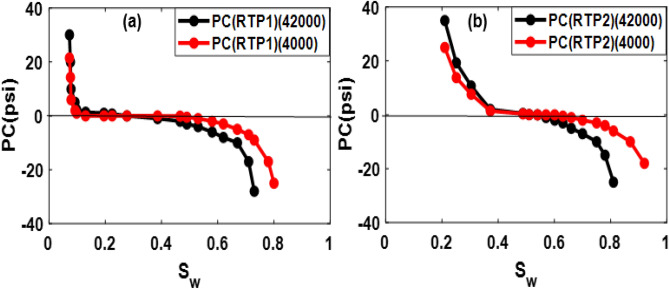
Comparison of HSW (42,000) and low-salinity water (4000 ppm) capillary pressure for RTP1 (**a**) and RTP2 (**b**).

The salinity condition of 4000 ppm is considered to determine averaged relative permeability and capillary pressure curves. For RTP1 and RTP2, the comparison of HSW (42,000) and LSW relative permeability curves is shown in Fig. 8a,b:

Comparison of HSW and LSW capillary pressure for RTP1 and RTP2 is as Fig. 9a,b:

## Discussion

As shown in Fig. 8a,b, as a result of changing salinity from high to low, water relative permeability curves have declined significantly, and oil relative permeability curves have increased. It is noticed that the change in water relative permeability appears to be higher than in the oil relative permeability. Moreover, the relative permeability curve shifted to the right, which implied higher water wettability. For RTP2, due to the clay content, a noticeable shift toward the highest saturations is observed. Moreover, a decrease in residual oil saturation and an increase in irreducible water saturation are also observed, which confirms the water-wetting nature of the rock.

As demonstrated in Fig. 9a,b, less negative capillary pressure is obtained by LSW flooding, and thereby, water flood efficiency is improved.

A shift in capillary pressure due to clay content is also observed in RTP2 compared to RTP1. Moreover, the capillary pressure of RTP2 has fewer negative parts due to its wetting nature. The obtained averaged low-salinity curves would be assigned to grid cells in the reservoir simulator to investigate the low-salinity flood process at the core and field (after upscaling) scale.

**Figure 10:**
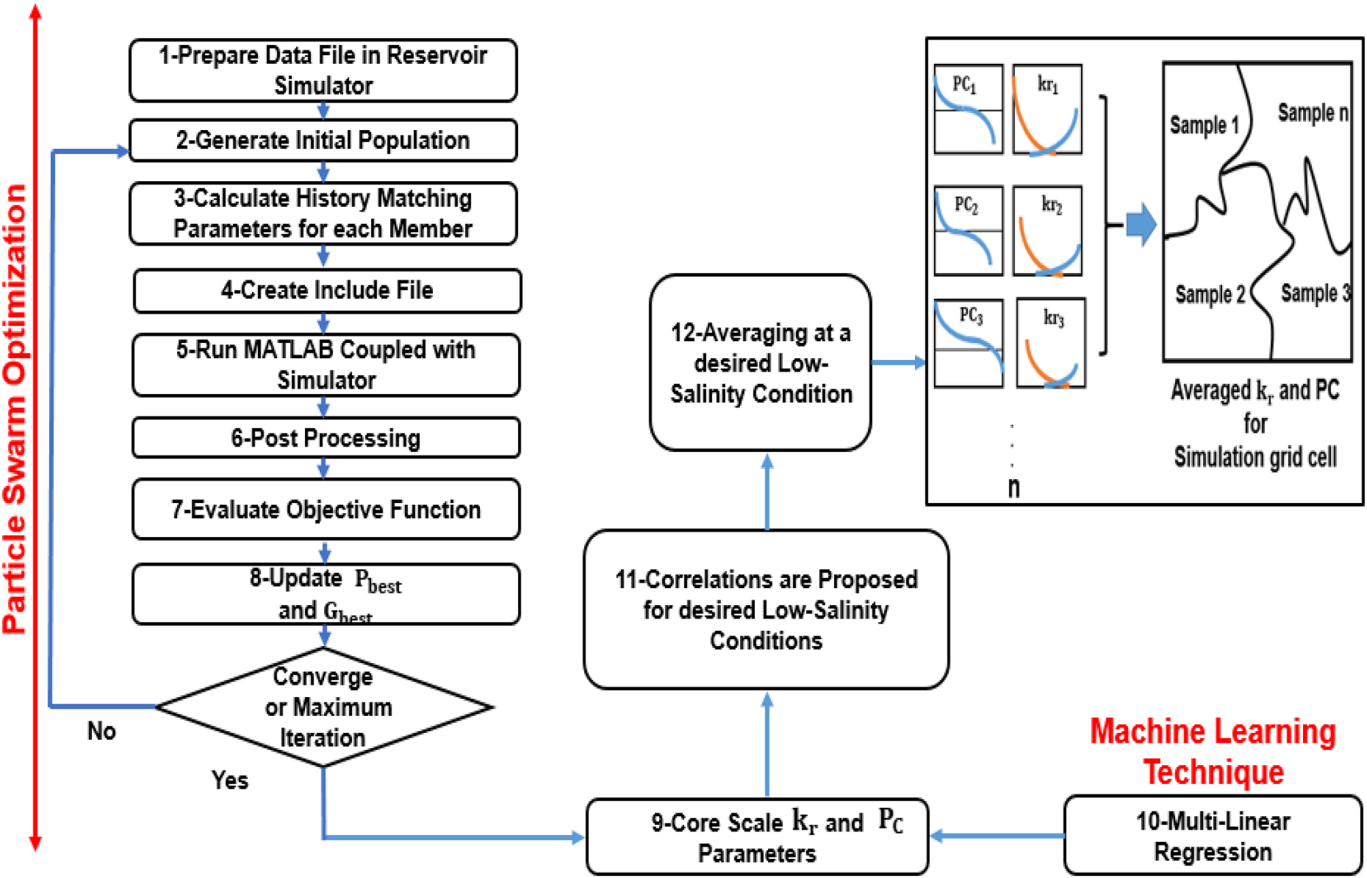
Hybrid particle swarm optimization with machine learning technique to achieve averaged relative permeability and capillary pressure curves at a desired low-salinity condition.

The entire process to achieve averaged relative permeability and capillary pressure curves at a desired low-salinity condition is shown in Fig. 10:

The proposed hybrid method of coupling PSO and machine learning technique for estimating relative permeability and capillary pressure functions could be a suitable tool for core scale simulations of water-based EOR methods, and in particular, low-salinity flooding applications. Moreover, upscaling the obtained averaged low-salinity curves would help investigate the low-salinity flood process at the field scale.

## Conclusions

Due to the challenges of obtaining the experimental data of relative permeability and capillary pressure in the low-salinity flood, hybrid particle swarm optimization coupled with a multiple linear regression method is proposed in this study to determine the relative permeability and capillary pressure functions. To this end, the particle swarm optimization algorithm is coupled with a simulator to provide the required data from previously lab-reported studies for low-salinity floods. By having the matching parameters in two different salinities (high and low), the relative permeability and capillary pressure parameters were estimated through linear interpolation in other low-salinity conditions, and finally, by using multi-linear regression, a set of correlations for relative permeability and capillary pressure functions for low salinity flood were developed. The experimental conditions for developing correlations included the porosity range of 0.15–0.4 for rock type 1, and 0.1–0.35 for rock type 2, the permeability range of 0.01–100 Darcy for rock type 1, and 0.00001–100 Darcy for rock type 2, type of wettability (mixed and weakly water wet), salinity range from 1000 to 10000 ppm, and clay content. The weighted-relative permeability approach and the Leverett J function were used as averaging techniques to assign one single curve of relative permeability and capillary pressure. The main results obtained in this study are as follows:

- As a result of automatic history matching by coupling particle swarm optimization algorithm and a simulator, relative permeability and capillary pressure parameters were obtained with less than 5% error and a running time of 2.5 h.
- The developed correlations were validated against experimental results of no clay rock type and clayey formation rock type with regression of 95% and 97%, and mean square error of 1.2E-04 and 8E-05 respectively.
- Based on the obtained averaged relative permeability and capillary pressure curves, the effect of salinity on the results was investigated. By changing salinity from 42000 to 4000 ppm, water relative permeability curves have declined significantly, and oil relative permeability curves have increased. The change in water relative permeability appears to be higher than oil relative permeability.
- The effect of clay content on the results was also investigated. For the clayey formation rock type, a noticeable shift toward the highest saturations related to the clay content in the relative permeability and capillary pressure curves was observed. Also, the corresponding capillary pressure curve exhibited fewer negative parts as compared to the no-clay rock type.
- The proposed hybrid method could be a suitable tool for core scale simulations of the water-based EOR methods. Upscaling the obtained averaged low-salinity curves would help investigate the low-salinity flood process at the field scale since limited data are available on the subject, which is suggested as future work.

## List of symbols

$${{\text{a}}}_{1.2}$$: Correlation parameters [−]
$${{\text{b}}}_{1}$$: Correlation parameters [−]
$${{\text{k}}}_{1.2}$$: Correlation parameters [−]
${{\text{J}}}_{{\text{ave}}}$: The average J-function for a block [−]
${{\text{J}}}_{({\text{Sw}})}$: The Leverett J function [−]
k: Absolute permeability [$${{\text{m}}}^{2}$$]
${{{\text{kr}}}_{{\text{a}}}}_{({\text{Ave}})}$: The phase average relative permeability [−]
${{{\text{kr}}}_{{\text{w}}/{\text{o}}}}_{({\text{HSW}})}^{{\text{end}}}$: Water/oil endpoint relative permeability at high salinity [−]
${{{\text{kr}}}_{{\text{w}}/{\text{o}}}}_{({\text{LSW}})}^{{\text{end}}}$: Water/oil endpoint relative permeability at low-salinity [−]
${{{\text{n}}}_{{\text{w}}/{\text{o}}}}_{({\text{HSW}})}$: The constant of Corey’s equation for water/oil at high salinity [−]
${{{\text{n}}}_{{\text{w}}/{\text{o}}}}_{({\text{LSW}})}$: The constant of Corey’s equation for water/oil at low-salinity [−]
${{{\text{P}}}_{\mathrm{C }}}_{{\text{ave}}}$: The average capillary pressure [kPa]
${({\text{RF}})}_{{\text{Exp}}}$: Measured recovery factor [−]
${({\text{RF}})}_{{\text{Sim}}}$: Simulated recovery factor [−]
${{\text{S}}}_{{\text{nw}}}$: Normalized water saturation [m^3^]
${{\text{Sor}}}_{({\text{HSW}})}$: Residual oil saturation at high salinity [m^3^/m^3^]
${{\text{Sor}}}_{({\text{LSW}})}$: Residual oil saturation at low-salinity [m^3^]
${{\text{T}}}_{{\text{x}}}$: The phase transmissibility [m^2^/mp s]
${{\text{V}}}_{{\text{i}}}$: The rock sample’s bulk volume [${{\text{m}}}^{3}$]
${(\Delta {\text{P}})}_{{\text{Exp}}}$: Measured differential pressure [kPa]
${(\Delta {\text{P}})}_{{\text{Sim}}}$: Simulated differential pressure [kPa

## Greek letters

θ: The fluid’s contact angle [−]
${\upmu }_{{\text{a}}}$: The phase viscosity [mPa·s]
$$\mathrm{\varphi }$$: Porosity [m^3^/m^3^]
σ: The interfacial tension [N/m
